# Prevalence and determinants of suboptimal health status among outdoor labor workers in Chengdu, Southwest China: A cross-sectional study

**DOI:** 10.1371/journal.pone.0338995

**Published:** 2026-05-15

**Authors:** Lanchuan Zhang, Xue Li, Junwei Wang, Ping Shao, Qiaojing Gao

**Affiliations:** 1 School of Sports Medicine and Health, Chengdu Sport University, Chengdu, China; 2 Sichuan Institute of Sports Science, Sichuan Anti-Doping Agency, Chengdu, China; Sichuan Agricultural University, CHINA

## Abstract

**Background:**

Suboptimal health status, termed sub-health, is a global public health problem. It is an intermediate health condition between health and disease. This study aimed to investigate the prevalence of chronic sub-health conditions among outdoor labor workers in Chengdu (a major city in Southwest China) and identify its associated risk factors.

**Methods:**

The Sub-Health Measurement Scale Version 1.0 (SHMS V1.0) was used to investigate the occurrence of sub-health conditions and their risk factors. Participants from four main outdoor occupations (drivers, construction workers, security guards, couriers) were surveyed to analyze the occurrence of sub-health and the associations between sub-health and other socio-demographic factors as well as exercise habits, using Pearson's correlation analysis and the multifactorial binary logistic regression model.

**Results:**

A total of 1030 questionnaires were distributed and 902 questionnaires were recovered (88% recovery rate). Excluding the invalid questionnaires due to omitted questions, multiple choices, etc., 705 questionnaires were retained (78% validity rate) and the prevalence of varying degrees of sub-health was 80.28%. Several significant correlating factors of the sub-health condition were identified, such as age, year of outdoor service, marital status, family size, monthly income, drinking habit, and regular participation in exercise.

**Conclusions:**

The incidence of subhealth was relatively high among outdoor workers, while no significant age-related difference was observed. The incidence also varies across the different occupations (*i.e.*, drivers > security guards > construction workers > couriers). Future targeted measures to alleviate these contributing factors may improve the sub-health status of outdoor labor workers.

## 1 Introduction

The World Health Organization (WHO) defines health as a state of complete physical, mental, and social well-being, transcending the mere absence of disease or infirmity [1]. In contrast, suboptimal health, often referred to as sub-health, represents an intermediate physical state between health and disease. Marked by chronic fatigue and mental distress persisting for over three months [2], sub-health can manifest in three distinct forms: physical, mental, and interpersonal sub-health [3]. While not a disease in itself, sub-health can give rise to symptoms such as non-specific pain (*e.g.*, chest and/or back pain), vertigo, headache, anxiety, and depression [4]. Moreover, it may act as a precursor for facilitating the development or exacerbation of chronic diseases. As suboptimal health has emerged as a significant global public health concern, yet its root causes remain largely elusive, there is an immediate imperative to conduct in-depth investigations on sub-health. Such explorations are crucial for formulating effective prevention and management strategies for chronic diseases and enhancing the overall well-being of individuals and communities.

Suboptimal health is currently a widespread issue, with reported prevalence rates of 65% among adolescents, 71% in middle-aged adults, and 72% in the elderly [5]. Previous research across diverse populations has consistently identified a correlation between psychological symptoms and sub-health [6–7]. Individuals suffering from anxiety disorders, for instance, not only experience intense fear and worry but also exhibit physical symptoms like sweating, trembling, dizziness, and rapid heartbeat, all of which take a toll on both their physical and mental health. A notable study has even established a strong link between sub-health and anxiety/depression [8].

With societal progress and evolving lifestyles, the problem of sub-health has become increasingly pronounced, attracting a growing body of investigations [9]. Previous studies have predominantly focused on high-risk groups such as students, healthcare workers, teachers, and civil servants [8–11]. However, these investigations have some limitations. For example, studies on students often focused on the stress of academic performance on suboptimal health, but rarely considered the influence of other life-style factors such as irregular diet caused by campus food choices and insufficient outdoor activities due to heavy study tasks. In research on healthcare workers, while the high-pressure working environment and long working hours were often mentioned, the impact of frequent night shifts on circadian rhythms and the psychological stress of dealing with life-and-death situations were not fully explored.

On the other hand, outdoor labor workers, who play a vital role in urban construction and service provision, are particularly susceptible to chronic fatigue, a common feature of sub-health [12]. This vulnerability is primarily attributable to their arduous working conditions and high-stress levels. For instance, in Chengdu, a city characterized by its humid subtropical climate, outdoor laborers face unique environmental challenges. The sweltering and humid summers can lead to heat-related illnesses, heatstroke, and dehydration, while the relatively mild but often cloudy winters may contribute to musculoskeletal discomfort due to the damp weather conditions. Additionally, Chengdu's rapid urbanization in the recent decades has spurred a surge in demand for outdoor labor, yet current occupational policies may not adequately safeguard the health of these workers. There are often no clear regulations on maximum working hours during extreme weather, and the provision of necessary health protection equipment is insufficient.

Against this backdrop, we put forward several explicit hypotheses. First, we hypothesize that outdoor labor workers in Chengdu will exhibit a relatively high prevalence of sub-health compared to the local general population. Second, we propose that several key factors such as long working hours (often exceeding 10 hours a day in construction projects), exposure to adverse environmental conditions (including intense sunlight in summer and cold winds in winter), and limited access to healthcare services (due to work-related constraints and financial limitations) will significantly contribute to their sub-health status.

The implications of suboptimal health among outdoor labor workers extend beyond individual well-being. It is closely associated with productivity loss. Workers in a sub-health state are more likely to take sick leave, experience reduced work efficiency, and make more mistakes on the job, all of which can disrupt project schedules and increase operational costs for employers. In terms of healthcare expenditure, the treatment of sub-health related symptoms and the potential development of chronic diseases stemming from it would impose a substantial financial burden on both workers and the healthcare system.

In addition, our current study has adopted a cross-sectional research model. By collecting data from a representative sample group of outdoor labor workers engaged in urban construction and daily life services in Chengdu, we can systematically investigate the prevalence, causes, and associated factors of sub-health. Through this approach, we aimed to offer actionable insights to healthcare providers, employers, and policymakers, with a goal of improving the health of outdoor labor workers and alleviating socio-economic burdens associated with suboptimal health in this crucial occupational group.

## 2 Methods

### 2.1 Participants

This study selected four types of outdoor labor workers, *i.e.*, drivers, couriers, security guards, and construction workers, due to their substantial contributions to urban development and the unique insights they may offer into the labor market dynamics. The city of Chengdu is a representative urban center in China, and the scale of these occupational groups is particularly striking. As of March 2025, the city reported 310,000 licensed ride-hailing drivers, and complementarily, data from 2024 indicated that approximately 75,000 food delivery couriers were actively operating, highlighting the increasing demand for on-demand delivery services. Meanwhile, official statistics in 2024 revealed over 120,000 registered security guards in the city, underscored their critical role in maintaining public safety. The construction sector, a cornerstone of urban infrastructure, employed an estimated workforce of ~1 million construction workers. These statistic figures not only underscore the sheer size of these occupational groups but also their integral roles in urban logistics, safety, and infrastructure development.

The recruitment period for participants was from August 1, 2021 to July 23, 2023. The human study protocol was approved by the Ethics Committee of Chengdu Sport University (Approval No. 2021−1，date of approval: January 14, 2021), and all outdoor labor workers from Chengdu voluntarily participated in this survey-based study. Every participant signed an informed consent form. To confirm the validity of the measurement tool – Sub-Health Measurement Scale V1.0 (SHMS V1.0), we verified that SHMS V1.0 was validated previously by the developers of the scale [3], who randomly selected a total of 15,066 names from six administrative regions in China (*i.e.*, North, South, East, Southwest, Northwest, and Northeast) for epidemiological investigations and constructed a sub-health assessment scale norm that is applicable to the Chinese region and also provides a reference basis for rapid screening and diagnosis of sub-health.

Subsequently, online questionnaires were distributed to the participating outdoor labor workers during the fieldwork using stratified whole cluster random sampling. The subjects were randomly selected for the questionnaire survey. The occupation was used as a stratifying factor in the outdoor labor workers, who were surveyed in four categories of occupational distribution, *i.e.*, construction workers, drivers, couriers, and security guards. A total of 1,030 questionnaires were distributed, and 902 questionnaires were recovered (88% recovery rate). After discounting the invalid questionnaires due to missing questions, multiple choices, etc., 705 valid questionnaires were retained (78% validity rate). The participants were not involved in the development of research questions or study design.

### 2.2 Inclusion and exclusion criteria

Inclusion criteria of the study participants include 1) Good cognitive status, no verbal communication disorder, and ability to fully cooperate with the research process; 2) Without the following diagnosed diseases that may cause symptoms: cardio-cerebral vascular diseases, digestive diseases, respiratory diseases, urinary diseases, endocrine system diseases, bone and joint diseases, gynecological diseases, and mental diseases; 3) Aged between 18 and 60 years old; and 4) Not receiving any treatment for fatigue, including taking dietary supplements, within 1 month prior to the study.

Exclusion criteria of the study participants include: 1) Suffering from an acute or chronic diagnosed disease; 2) Experiencing major negative events within the past six months (e.g., demotion or disciplinary action, laid off, break-up of relation, divorce, serious illness or loss of a relative or close friend, household financial problems, loss of valuable belongings, overly stress at work, legal dispute, traffic accident, etc.; 3) Having other known causes of fatigue, such as post-cancer fatigue and other conditions causing severe fatigue; and 4) Unwilling to cooperate with the survey or participating in another study that may affect the results of this study.

### 2.3 Questionnaire design, measurements, and validation

SHMS V1.0 was used to assess the sub-health condition. The questionnaire was formulated through rigorous Delphi expert consultation, item analysis, item screening, etc. to formulate the “Sub-Health Quantitative Measurement Scale” that conforms to the special cultural background and values of the Chinese population. The questionnaire was divided into three parts: 1) General information about the outdoor labor workers; 2) Factors related to sub-health status; and 3) Exercise habits. The general information included gender, age, occupation, etc. The sub-health-related factors included education, marriage, family, income, etc. Exercise habits included frequency and time of exercise. Each question on the questionnaire can only choose one of the options as the answer and multiple choices and/or omissions were listed as invalid options. When the invalid option reached 20%, it was considered an invalid questionnaire. This questionnaire was previously tested by a large sample of people for its good reliability and validit [13]. The structure of the questionnaire scale is summarized in Table 1.

**Table 1:** Structure of sub-health rating scale 1.0 scale.

Perspective	Dimensionality	N	Item distribution
**Physical health**	Physical symptom（P1）	3	1、2、3
	Organ function（P2）	6	4、5、6、7、8、9
	movement function（P3）	3	10、11、12
	energies（P4）	2	13、14
**Mental health**	positive emotion（M1）	4	16、17、18
	psychological symptom（M2）	6	20、21、22、23、24、25
	Cognitive function（M3）	2	26、27
**Social health**	Social adaptive（S1）	4	29、30、31、32
	Social resources and support（S2）	5	33、34、35、36、37
**Overall assessment**	Overall Evaluation Indicators for Sub-Health	4	15、28、38、39

The scale was scored on a five-point Likert scale ranging from 1–5. Forward scoring entries included questions 1–3, 13–19, and 26–39, their scores were the same as the raw scores, 1–5; reverse scoring entries included questions 4–12 and 20–25, and their scores were equal to 6 minus the raw scores, and the raw scores of the dimensions, subscales, and scales were finally converted into percentage scores. The specific scoring criteria are shown in Table 2.

**Table 2:** The norms of sub‑health measurement scale version 1.0 for Chinese ^[^13^]^.

Dimension	Gender	Age (year)	Disease	Severe sub-health	Moderate sub-health	Mild sub-health	Health
**GS**	**Male**	14 ~ 19	(0,57.89)	(57.89,64.16)	(64.16,76.7)	(76.7,82.96)	(82.96,100)
		20 ~ 29	(0,56.64)	(56.64,62.47)	(62.47,74.13)	(74.13,79.96)	(79.96,100)
		30 ~ 49	(0,55.85)	(55,85,62.19)	(62.19,74.85)	(74.85,81.18)	(81.18,100)
		50 ~ 64	(0,54.98)	(54.98,61)	(61,73.05)	(73.05,79.08)	(79.08,100)
		≥65	(0,52.89)	(52,89,59.16)	(59.16,71.7)	(71.7,77.97)	(77.97,100)
	**Female**	14 ~ 19	(0,55.21)	(55.21,61.54)	(61.54,74.21)	(74.21,80.55)	(80.55,100)
		20 ~ 29	(0,56.01)	(56.01,61.76)	(61.76,73.24)	(73.24,78.99)	(78.99,100)
		30 ~ 49	(0,54.95)	(54.95,60.99)	(60.99,73.05)	(73.05,79.09)	(79.09,100)
		50 ~ 64	(0,54.37)	(54.37,60.37)	(60.37,72.37)	(72.37,78.37)	(78.37,100)
		≥65	(0,51.71)	(51,71,57.93)	(57.93,70.36)	(70.36,76.57)	(76,57,100)
**PS**	**Male**	14 ~ 19	(0,63.33)	(63,33,69.67)	(69.67,82.36)	(82.36,88.7)	(88.7,100)
		20 ~ 29	(0,61.35)	(61.35,67.4)	(67.4,79.5)	(79.5,85.55)	(85,55,100)
		30 ~ 49	(0,58.75)	(58.75,65.39)	(65.39,78.65)	(78.65,85.28)	(85.28,100)
		50 ~ 64	(0,57.21)	(57,21,63.53)	(63,53,76.16)	(76.16,82.47)	(82,47,100)
		≥65	(0,53.67)	(53,67,60.85)	(60.85,75.19)	(75.19,82.37)	(82.37,100)
	**Female**	14 ~ 19	(0,60.74)	(60.74,66.98)	(66.98,79.46)	(79.46,85.7)	(85.7,100)
		20 ~ 29	(0,60.45)	(60.45,66.62)	(66,62,78.95)	(78.95,85.11)	(85.11,100)
		30 ~ 49	(0,57.91)	(57.91,64.32)	(64.32,77.12)	(77.12,83.52)	(83,52,100)
		50 ~ 64	(0,55.93)	(55,93,62.33)	(62.33,75.11)	(75.11,81.5)	(81.5,100)
		≥65	(0,51.25)	(51.25,58.63)	(58.63,73.37)	(73.37,80.75)	(80.75,100)
**MS**	**Male**	14 ~ 19	(0,52.13)	(52.13,59.86)	(59,86,75.34)	(75.34,83.07)	(83,07,100)
		20 ~ 29	(0,52.24)	(52.24,59.49)	(59.49,74)	(74,81.25)	(81,25,100)
		30 ~ 49	(0,53.4)	(53.4,60.92)	(60.92,75.96)	(75.96,83.48)	(83.48,100)
		50 ~ 64	(0,52.38)	(52,38,60.01)	(60.01,75.27)	(75,27,82.9)	(82.9,100)
		≥65	(0,51.63)	(51,63,58.99)	(58.99,73.72)	(73.72,81.08)	(81.08,100)
	**Female**	14 ~ 19	(0,49.76)	(49,76,57.47)	(57.47,72.88)	(72.88,80.59)	(80.59,100)
		20 ~ 29	(0,50.47)	(50,47,57.41)	(57.41,71.29)	(71.29,78.23)	(78.23,100)
		30 ~ 49	(0,51.13)	(51.13,58.54)	(58.54,73.35)	(73.35,80.75)	(80.75,100)
		50 ~ 64	(0,51.34)	(51.34,58.8)	(58.8,73.73)	(73.73,81.19)	(81.19,100)
		≥65	(0,49.14)	(49.14,56.5)	(56.5,71.21)	(71,21,78.57)	(78.57,100)
**SS**	**Male**	14 ~ 19	(0,48.43)	(48,43,56.97)	(56.97,74.04)	(74.04,82.58)	(82,58,100)
		20 ~ 29	(0,46.63)	(46.63,54.5)	(54.5,70.24)	(70.24,78.11)	(78.11,100]
		30 ~ 49	(0,46.81)	(46,81,54.99)	(54.99,71.35)	(71.35,79.53)	(79.53,100)
		50 ~ 64	(0,45.87)	(45.87,53.85)	(53.85,69.81)	(69,81,77.79)	(77,79,100)
		≥65	(0,42.73)	(42,73,51.44)	(51.44,68.88)	(68,88,77.59)	(77,59,100)
	**Female**	14 ~ 19	(0,46)	(46,54.59)	(54.59,71.77)	(71.77,80.36)	(80,36,100)
		20 ~ 29	(0,48.56)	(48.56,56.02)	(56.02,70.95)	(70.95,78.41)	(78.41,100)
		30 ~ 49	(0,47.6)	(47.6,55.15)	(55.15,70.26)	(70,26,77.81)	(77,81,100)
		50 ~ 64	(0,47.3)	(47.3,55.08)	(55.08,70.63)	(70.63,78.41)	(78.41,100)
		≥65	(0,45.57)	(45.57,53.6)	(53.6,69.67)	(69.67,77.71)	(77.71,100)

The reliability of the scale was tested to determine the consistency and stability of the results of this questionnaire and to reduce random errors. The construct reliability of the scale was also tested to clarify that the questionnaire was able to measure the various constructs assumed by the designers of the questionnaire. The results of the reliability test can be found in the S1 Table–S3 Table.

### 2.4 Survey methodology and quality control

Once the sampling scheme was finalized, a standardized survey guide was employed to train survey assistants in each designated survey area. Local survey assistants received training at their respective sampling points and collaboratively oversaw the survey process. Prior to commencing the survey, a comprehensive and detailed explanation was provided to all the respondents, outlining the survey's purpose, significance, confidentiality of the survey results, and the methodology for obtaining information. This ensured the respondents possessed a clear understanding of the survey's objectives, significance, benefits, and potential risks.

For survey participants capable of independently completing the questionnaire, the investigators provided guidance as needed. However, for respondents who were unable to independently complete the survey, due to reasons such as visual impairment or old age, the investigators objectively read out the questions and response options to the respondents and recorded the respondents’ choices/answers on their behalf.

During the survey, a rigorous approach was maintained, including retrieval of the completed questionnaires and on-site quality checks. In cases where a significant number of respondents have omitted responses, they are encouraged to complete the questionnaire on the spot. Upon conclusion of the survey, the retrieved questionnaires underwent a systematic screening process. Questionnaires with a completion rate below 80%, those that exhibited poor quality responses (such as providing the same answer for all questions, multiple choices, and omissions), or any instances of two questionnaires with identical responses were excluded. A qualitative assessment and verification were conducted as a part of this screening process.

### 2.5 Statistics

After screening out the valid questionnaires, Microsoft Excel was used for data entry and processing. SPSS 26.0 software was used to analyze the collected data. Descriptive analysis, χ² test, independent t-test, and multifactor binary logistic regression model were used for statistical analysis of the data.

## 3 Results

### 3.1 Incidence of sub-health

**Fig 1:**
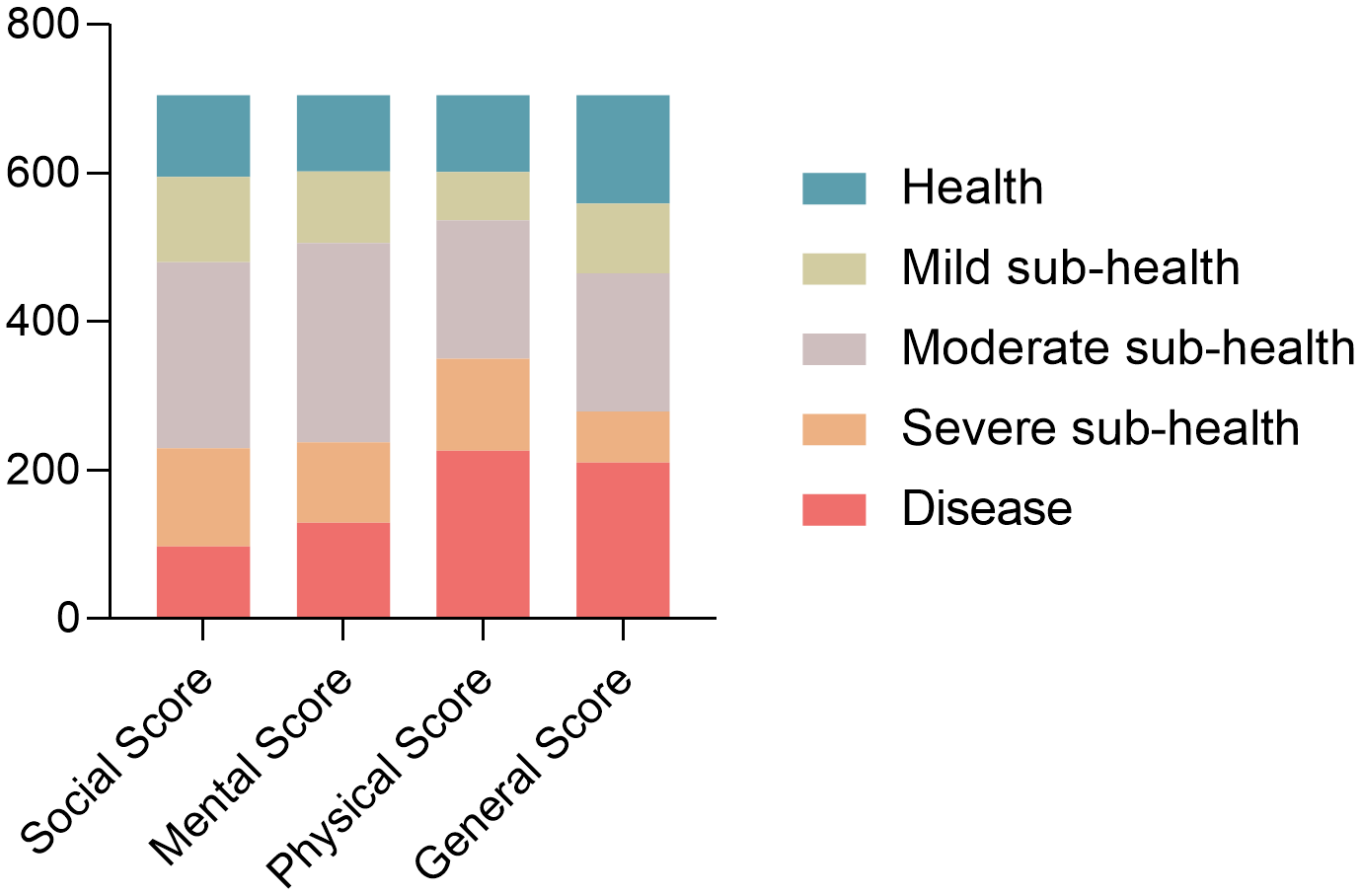
The overall incidence of sub-health in outdoor labor workers. This bar chart illustrates the distribution of the individuals across 5 health states: Disease, Severe Sub-health, Moderate Sub-health, Mild Sub-health, and Health, based on 4 different quantitative scores: Social Score, Mental Score, Physical Score, and General Score. The number Y-axis represents the number of participants in each health state category.

A total of 1030 questionnaires were distributed and 902 questionnaires were recovered, with a recovery rate of 88%. Discounting the invalid questionnaires (omitted questions, multiple choices, etc.), 705 valid questionnaires were retained, with a validity rate of 78%. Among them, 146 were categorized as “Health,” 94 as “Mild sub-health,” 186 as “Moderate sub-health,” 69 as “Severe sub-health,” and 210 as “Disease”(as shown in Fig 1).

The relatively lower overall sub-health rates among the studied outdoor labor workers can be attributed to multiple factors. Firstly, the occupational characteristics of outdoor laborers may play a role. Although the work is physically demanding, it also provides regular physical exercise. Continuous physical activity in the course of work helps to maintain muscle strength, improve cardiovascular function, and enhance the body's overall immunity, thereby reducing the risk of sub-health. Secondly, the unique social environment in Chengdu has a positive impact. Community-based health education programs have been widely carried out, raising workers’ awareness of health maintenance. Workers are more likely to adopt healthy behaviors, such as balanced diets and regular sleep patterns. Moreover, some employers in Chengdu have started to implement more human-centered management strategies, including providing appropriate rest breaks, improving workplace safety and comfort, and offering health-related benefits. These measures collectively contribute to reducing the stress levels of workers and promoting their overall well-being, ultimately leading to a lower prevalence of sub-health in the current study population.

### 3.2 Age-dependent incidence of sub-health

**Fig 2:**
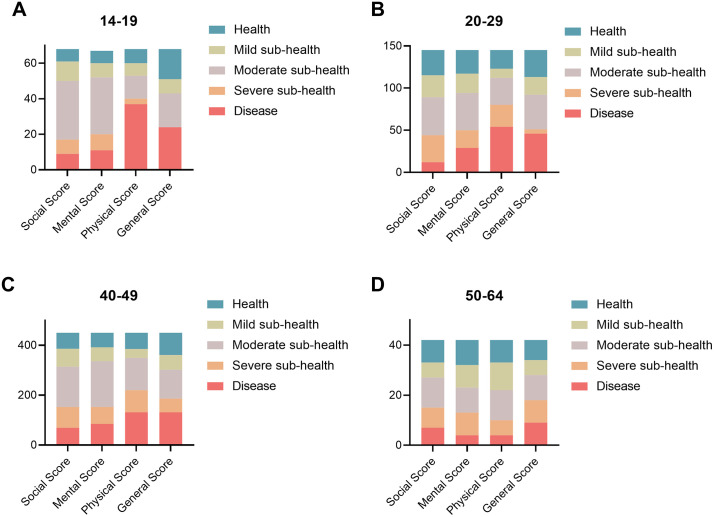
Incidence of sub-health by the age of outdoor labor workers. The proportion of sub-health status is plotted under the following 4 age groups: A) 10-19 years; B) 20-29 years; C) 30-49 years; D) 50-64 years;. The data are categorized into 5 health states, i.e., Severe Sub-health, Moderate Sub-health, Mild Sub-health, and Health. The number Y axis represents the number of outdoor worker participants in each health state category.

Chi‑square test analysis indicated no significant difference in the prevalence of sub‑health among different age groups (χ² = 1.58, P = 0.664). Among the 705 valid survey responders, 57 participants were aged 10–19 years and 47 of them had sub-health status (sub-health incidence 82.46%). 132 were 20–29 years old and 101 of them had sub-health (sub-health incidence 76.52% OR=1.44, 95% CI:0.65–3.19). Among the 460 outdoor labor workers aged 30–49 years, 372 were in sub-health (sub-health incidence 80.87% OR=1.30, 95% CI: 0.82–2.06), whereas 56 out of 56 workers aged 50–64 years were sub-health (sub-health incidence 82.14% OR=1.41, 95% CI: 0.64, 3.12). Overall, the prevalence of sub-health remained at a high level of over 80% across all age groups, with only the 20–29 age group showing a slightly lower rate (as shown in Fig 2).

Although the overall prevalence of sub-health among outdoor manual workers in this study was as high as 80.28%, suggesting a relatively high baseline health risk in this population, age, as a continuous variable, showed no significant correlation with sub-health status.This finding may be attributed to the homogenizing effect of the occupational environment: regardless of age differences, all outdoor workers were consistently exposed to harsh natural conditions such as high temperature, strong sunlight, wind and rain, as well as multiple occupational risk factors including high-intensity physical workload, irregular work-rest schedules, and limited labor protection.The impact of these common adverse exposures on sub-health was far stronger than the physiological differences associated with age. Consequently, the potential incremental health risk across different age groups was masked by the overall high-risk working environment, leading to the absence of a significant association between age and sub-health.

The relatively low prevalence of sub-health among workers aged 20–29 years may be attributed to their stronger health awareness and better learning adaptability, which motivate them to actively adopt targeted protective measures—such as the proper use of personal protective equipment and maintenance of reasonable rest schedules—to reduce the risk of occupational injury. In contrast, workers aged 30 years and older usually develop fixed working habits through years of outdoor labor, accompanied by relatively weak health protection awareness. In addition, some older workers may neglect rest and health management due to financial pressure or heavy workloads, thereby further increasing their risk of sub-health.

### 3.3 Gender effects on incidence of sub-health

Sex at birth was used for gender classification. Among the participants, 625 were male, of whom 497 had sub-health, with a sub-health prevalence of 79.52%; 80 were female, of whom 62 had sub-health, with a sub-health prevalence of 77.5%.There was no significant difference in the prevalence of sub-health between males and females (χ² = 0.18, P = 0.67).

### 3.4 Incidence of sub-health in different occupations

**Fig 3:**
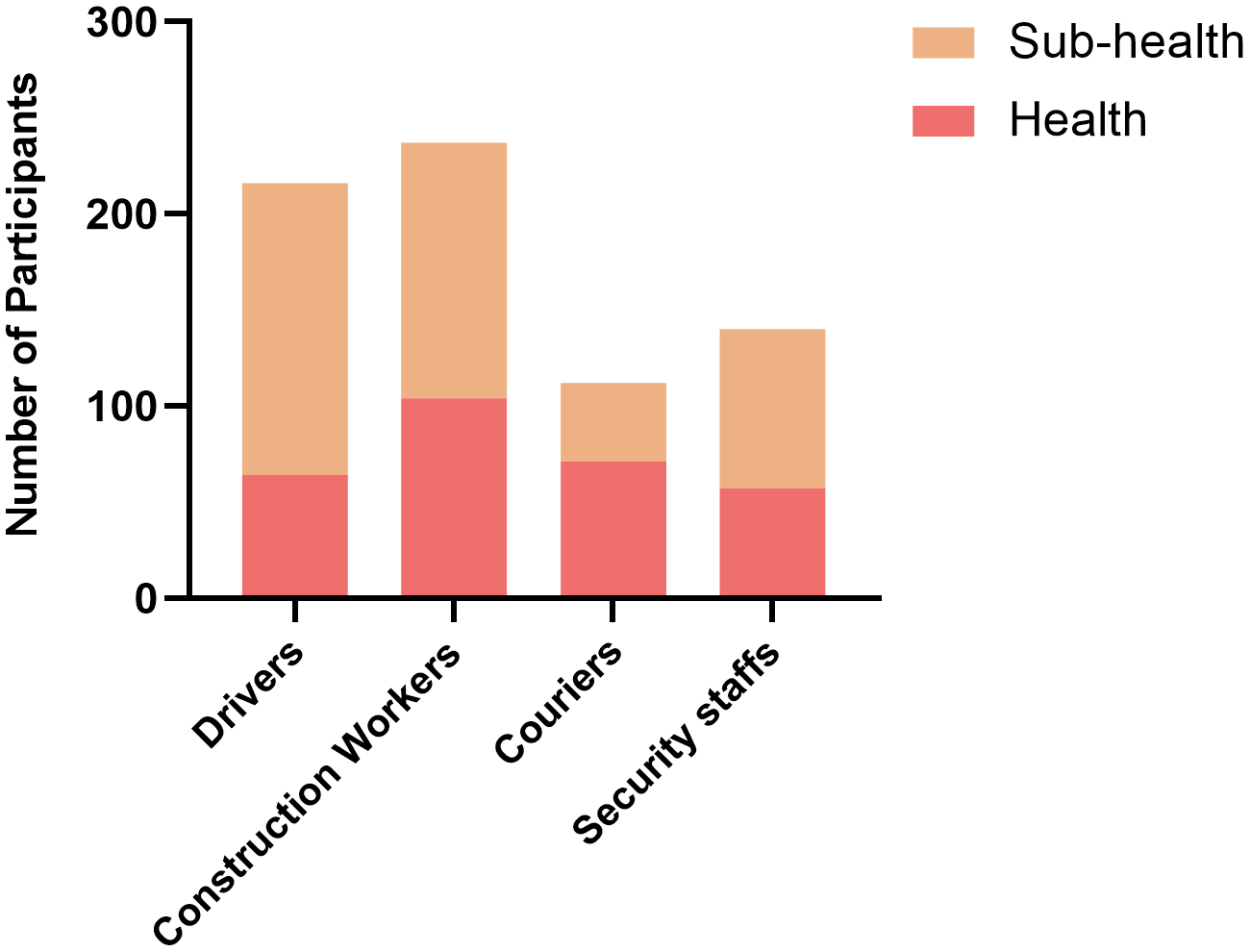
Occurrence of sub-health by occupation of the outdoor labor workers. The occurrence ratio of Sub-health versus Health among the outdoor labor workers in 4 occupations (i.e., drivers, construction workers, couriers, and security staffs). The number of Y axis represents the number of outdoor worker participants in Sub-heal th or Health category.

There was a significant difference in health status among different occupations (χ² = 35.05, P < 0.001). Drivers had the highest sub-health rate, while couriers had the lowest.Of 216 participants identified themselves as drivers, 152 reported suboptimal health (incidence: 70.37%, 95% CI: 0.64–0.76), whereas in 237 construction workers, 133 of them were in sub-health (incidence: 56.12%, 95% CI: 0.53–0.66). Among 112 couriers, 41 reported suboptimal health (incidence: 36.61%, 95% CI: 0.27–0.45). Lastly, in 140 security guards, 83 reported sub-health status (incidence: 59.29%, 95% CI: 0.52–0.674). Therefore, the drivers exhibited the highest prevalence of sub-health incidence, followed by the security guards, the construction workers, and the couriers with the lowest prevalence (Fig 3).

### 3.5 Correlations between the variables

The overall health level is treated as dependent variable (1 = healthy, 2 = sub-health status), whereas factors including age, gender, and educational background are treated as independent variables in binary logistic regression analysis. Table 3 shows the regression analysis results.

**Table 3:** Binary logistic regression analysis factors associated with overall sub-health of outdoor manual workers.

Risk factors	B	S.E.	Wald	df	Sig.	OR	95%C.I.for EXP(B)	
							Lower	Upper
Age	0.017	0.013	1.664	1	0.197	1.017	0.991	1.043
Gender	0.661	0.398	2.755	1	0.097	1.937	0.887	4.226
Years of service	−0.299	0.111	7.315	1	0.007	0.741	0.597	0.921
Education	−0.021	0.15	0.02	1	0.889	0.979	0.73	1.313
Marital status	−0.331	0.301	1.208	1	0.272	0.718	0.398	1.296
Family size	0.199	0.119	2.803	1	0.094	1.22	0.967	1.54
Monthly income	−0.534	0.199	7.211	1	0.007	0.586	0.397	0.866
Current Address	−0.102	0.249	0.17	1	0.68	0.903	0.555	1.469
Smoking	0.279	0.16	3.053	1	0.081	1.322	0.967	1.809
Drink	−0.336	0.254	1.743	1	0.187	0.715	0.434	1.177
Medical History	−0.129	0.207	0.386	1	0.534	0.879	0.585	1.32
Interest in sports	−0.572	0.257	4.953	1	0.026	0.565	0.341	0.934
Regular Attendance	−0.208	0.278	0.561	1	0.454	0.812	0.472	1.399
Reasons for non-participation	0.136	0.091	2.228	1	0.136	1.145	0.958	1.369
Exercise Recognition	0.627	0.215	8.471	1	0.004	1.872	1.227	2.854
Exercise plan	0.004	0.006	0.66	1	0.417	1.004	0.994	1.015
Exercise purpose	−0.12	0.144	0.689	1	0.406	0.887	0.669	1.177
Exercise frequency	0.008	0.086	0.009	1	0.924	1.008	0.853	1.192
Exercise Time period	0.156	0.166	0.891	1	0.345	1.169	0.845	1.617
Exercise duration	0.047	0.108	0.188	1	0.664	1.048	0.848	1.295
Exercise location	0.025	0.219	0.013	1	0.909	1.025	0.667	1.576
Exercise Companionship	0.026	0.278	0.009	1	0.926	1.026	0.595	1.77
Exercise Effect	0.017	0.013	1.664	1	0.197	1.017	0.991	1.043

**Fig 4:**
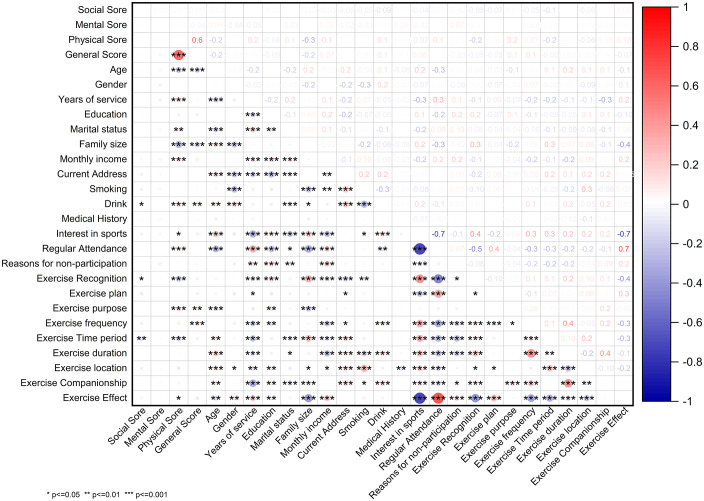
Analysis of the factors related to overall sub-health status of the outdoor labor workers. This graph summarizes the correlation analysis among the various risk factors of sub-health status. Each cell contains a correlation score between two variables listed in the corresponding row and column, in which a larger area of the circle represents a higher correlation score, whereas the color of circles is to distinguish a positive versus negative correlation. The right color bar indicates the correlation coefficient. Symbols in cells indicate the statistical significance of correlations: *p<=0.05, **p<=0.01, *** p<=0.001.

Based on the clinical manifestations of sub-health and the risk perception sample data, we calculated Pearson correlations between the study variables. There were various correlations between the sub-health and personal conditions, lifestyle habits, and exercise habits (as shown in Fig 4).

## 4 Discussion

To our best knowledge, the present study is the first attempt to investigate the sub-health status of the Chinese urban outdoor labor workers and therefore yielded some first-hand novel findings. The high prevalence of sub-health among outdoor manual workers is mainly driven by unavoidable harsh environmental exposure and high-intensity physical workload [14]. Prolonged exposure to extreme weather, air pollution, noise, and dust causes chronic damage to the respiratory, cardiovascular, and integumentary systems [15]. Meanwhile, long-term repetitive movements and heavy loading induce persistent musculoskeletal strain, which is a typical manifestation of sub-health. Irregular working hours also disrupt sleep and weaken immune function, further aggravating sub-health status.

The sustained physical exertion of outdoor work exceeds normal physiological limits, leading to an imbalance between energy consumption and recovery [16]. Insufficient nutritional intake and inadequate rest due to long commutes and family responsibilities prevent timely repair of muscle and neural fatigue. With increasing work experience, age-related physiological decline further intensifies this imbalance and maintains a high risk of sub-health.

Outdoor manual workers also face disadvantages in health resource access and self-management. Limited social security, insufficient workplace health support, and irregular lifestyles contribute to poor health outcomes [17]. Moreover, low health awareness leads to delayed intervention against early sub-health symptoms, allowing progressive deterioration.

On the other hand, economic and social pressure can also accelerate the occurrence of sub-health. The outdoor labor workers in China generally received lower wages and were more susceptible to financial difficulties. Their lower social-economic status may limit their access to nutritious diet and regular exercise that can impact the occurrence of sub-health as well as the timely symptomatic treatment. As shown in Fig 3, the work characteristics of different occupations also have a significant impact on sub-health. First, the most obvious common feature between drivers and security guards is a sedentary lifestyle that leads to the highest incidence of sub-health among the surveyed outdoor occupations. Growing evidence indicates that sedentary behavior may be associated with increased cardiovascular and overall mortality, since insufficient physical activity predicts premature death and the burden of cardiovascular disease [18]. Secondly, the workers in these two occupations (drivers and security guards) do not have fixed working hours and often work for a long hours and stay late. When circadian rhythm is disrupted, it would lead to dysregulation of gut microbial homeostasis, which plays an important role in the regulation of digestive [19–22], immune [23–26], and nervous systems [27–30]. Circadian clock disruption also increases the risks of prostate, breast, ovarian, and colorectal cancer [31–34], representing a serious burden in public and occupational health. In addition, working in environment with unhealthy air quality (such as working in dust-exposed conditions) is associated with higher risk of chronic and/or end-stage renal disease [34]. The prolonged exposure to heavy physical activity in hot conditions in the outdoor labor workers could also increase the risk of heat-related illnesses (e.g., heat stroke and heat exhaustion), cognitive decline, injury and death [35–37].

Furthermore, among the 15 factors analyzed as independent variables for the prevalence of sub-health in outdoor labor workers, the multi-categorical logistic regression analysis outdoor labor workers showed that age, year of service, marital status, family size, monthly income, drinking habit, interest in exercise, regular attendance of exercise, exercise purpose, exercise frequency, and exercise time period, etc. all affected the sub-health status of the urban outdoor labor workers in Southwest China. Apart from the uncontrollable biological factors such as age and gender, some controllable lifestyle factors including alcoholism and exercise habits can be used as an entry point to improve the sub-health status of outdoor labor workers.

Nonetheless, this study has several limitations. Firstly, a selection bias may have occurred during the participant recruitment process. Although we aimed to recruit a representative sample of outdoor labor workers in Chengdu, the participants were mainly from urban areas, potentially excluding those from rural or remote construction sites. This could lead to an overestimation or underestimation of the true prevalence of suboptimal health status among the entire outdoor labor workforce. Secondly, measurement bias might exist due to the reliance on self-reported questionnaires. Participants may have provided inaccurate information regarding their working hours, health status, and lifestyle habits, either due to forgetfulness or social desirability. To mitigate this, we ensured the questionnaires were clear and simple, but the complete elimination of such bias was challenging. Future studies could consider using objective measurement tools, such as wearable devices to monitor physical activity and physiological parameters, to enhance the accuracy of data collection.

## 5 Conclusion

The present study reveals that the health condition of urban outdoor labor workers in China is far from optimal, with a notable prevalence of suboptimal health status. To enhance the health of outdoor labor workers, several actionable steps should be implemented. Employers should be required to provide regular health check-ups and subsidized medical insurance for outdoor laborers, especially those in high-risk occupations like drivers and security guards, who exhibit relatively higher sub-health risks. Additionally, local authorities should enforce regulations mandating employers to ensure proper rest breaks, supply protective gear, and maintain safe working environments. Community organizations can play a role by organizing free health education programs focusing on stress management, healthy diet, and regular exercise for outdoor workers.

However, this study has limitations. The cross-sectional design restricts causal inference, and the small sample size of some subgroups may affect the generalizability of the findings. Future research with longitudinal designs and a larger number of samples, along with the use of objective measurement tools (such as wearable devices) to monitor physical activity and physiological parameters is needed to validate the overall conclusions of the current study.

## Supporting information

S1 Table Reliability analysis of the scale (N = 40 items). Cronbach’s α = 0.961, indicating excellent internal consistency.
(DOCX)

S2 Table Validity test results of the scale. KMO = 0.683; Bartlett’s sphericity test: χ² = 4924.077, df = 325, Sig. = 0.000, suitable for factor analysis.
(DOCX)

S3 Table Construct validity analysis of the scale. Nine common factors were extracted by principal component analysis, with a cumulative variance contribution rate of 77.529%.
(DOCX)
