# Supplementary material for: Prevalence and determinants of suboptimal health status among outdoor labor workers in Chengdu, Southwest China: A cross-sectional study
Source: PLoS One. 2026 May 15;21(5):e0338995. doi: 10.1371/journal.pone.0338995 (PMC13178926; doi:10.1371/journal.pone.0338995)
Supplement: S1 Table — Cronbach’s α = 0.961, indicating excellent internal consistency. (DOCX) [file pone.0338995.s001.docx]

**Supplementary Table 1**

Reliability analysis of the scale

| Cronbach's alpha | N of Items |
| --- | --- |
| 0.961 | 40 |
