# Supplementary material for: Prevalence and determinants of suboptimal health status among outdoor labor workers in Chengdu, Southwest China: A cross-sectional study
Source: PLoS One. 2026 May 15;21(5):e0338995. doi: 10.1371/journal.pone.0338995 (PMC13178926; doi:10.1371/journal.pone.0338995)
Supplement: S2 Table — KMO = 0.683; Bartlett’s sphericity test: χ² = 4924.077, df = 325, Sig. = 0.000, suitable for factor analysis. (DOCX) [file pone.0338995.s002.docx]

**Supplementary Table 2**

Results of validity tests

| KMO and Bartlett's test | | |
| --- | --- | --- |
| KMO | | .683 |
| Bartlett sphericity test | X^2^ | 4924.077 |
|  | DF | 325 |
|  | Sig | .000 |
