# Supplementary material for: Prevalence and determinants of suboptimal health status among outdoor labor workers in Chengdu, Southwest China: A cross-sectional study
Source: PLoS One. 2026 May 15;21(5):e0338995. doi: 10.1371/journal.pone.0338995 (PMC13178926; doi:10.1371/journal.pone.0338995)
Supplement: S3 Table — Nine common factors were extracted by principal component analysis, with a cumulative variance contribution rate of 77.529%. (DOCX) [file pone.0338995.s003.docx]

**Supplemental Table 3**

Results of the construct validity analysis of the scale

|  | Initial Eigen values | | | Extraction Sum of Squared Loadings | | | Rotation Sum of Squared Loadings | | |
| --- | --- | --- | --- | --- | --- | --- | --- | --- | --- |
| Component | Total | Variance | Cumulative | Total | Variance | Cumulative | Total | Variance | Cumulative |
| 1 | 16.898 | 42.246 | 42.246 | 16.898 | 42.246 | 42.246 | 8.412 | 21.030 | 21.030 |
| 2 | 2.854 | 7.135 | 49.380 | 2.854 | 7.135 | 49.380 | 4.935 | 12.338 | 33.368 |
| 3 | 2.433 | 6.082 | 55.462 | 2.433 | 6.082 | 55.462 | 3.837 | 9.593 | 42.961 |
| 4 | 2.094 | 5.235 | 60.698 | 2.094 | 5.235 | 60.698 | 3.397 | 8.492 | 51.453 |
| 5 | 1.694 | 4.235 | 64.933 | 1.694 | 4.235 | 64.933 | 2.930 | 7.325 | 58.778 |
| 6 | 1.487 | 3.717 | 68.650 | 1.487 | 3.717 | 68.650 | 2.037 | 5.093 | 63.872 |
| 7 | 1.393 | 3.481 | 72.131 | 1.393 | 3.481 | 72.131 | 1.973 | 4.934 | 68.805 |
| 8 | 1.096 | 2.740 | 74.872 | 1.096 | 2.740 | 74.872 | 1.815 | 4.538 | 73.344 |
| 9 | 1.063 | 2.657 | 77.529 | 1.063 | 2.657 | 77.529 | 1.674 | 4.185 | 77.529 |
| 10 | .866 | 2.166 | 79.695 |  |  |  |  |  |  |
| 11 | .827 | 2.066 | 81.761 |  |  |  |  |  |  |
| 12 | .760 | 1.901 | 83.662 |  |  |  |  |  |  |
| 13 | .723 | 1.806 | 85.468 |  |  |  |  |  |  |
| 14 | .695 | 1.737 | 87.205 |  |  |  |  |  |  |
| 15 | .587 | 1.468 | 88.673 |  |  |  |  |  |  |
| 16 | .502 | 1.256 | 89.929 |  |  |  |  |  |  |
| 17 | .489 | 1.221 | 91.151 |  |  |  |  |  |  |
| 18 | .478 | 1.195 | 92.346 |  |  |  |  |  |  |
| 19 | .398 | .995 | 93.341 |  |  |  |  |  |  |
| 20 | .381 | .953 | 94.294 |  |  |  |  |  |  |
| 21 | .323 | .809 | 95.103 |  |  |  |  |  |  |
| 22 | .271 | .678 | 95.780 |  |  |  |  |  |  |
| 23 | .240 | .601 | 96.381 |  |  |  |  |  |  |
| 24 | .203 | .507 | 96.888 |  |  |  |  |  |  |
| 25 | .181 | .452 | 97.340 |  |  |  |  |  |  |
| 26 | .166 | .416 | 97.755 |  |  |  |  |  |  |
| 27 | .157 | .392 | 98.147 |  |  |  |  |  |  |
| 28 | .135 | .339 | 98.486 |  |  |  |  |  |  |
| 29 | .113 | .282 | 98.768 |  |  |  |  |  |  |
| 30 | .105 | .263 | 99.031 |  |  |  |  |  |  |
| 31 | .078 | .194 | 99.225 |  |  |  |  |  |  |
| 32 | .068 | .171 | 99.396 |  |  |  |  |  |  |
| 33 | .062 | .156 | 99.552 |  |  |  |  |  |  |
| 34 | .047 | .119 | 99.670 |  |  |  |  |  |  |
| 35 | .043 | .108 | 99.778 |  |  |  |  |  |  |
| 36 | .031 | .078 | 99.856 |  |  |  |  |  |  |
| 37 | .026 | .065 | 99.921 |  |  |  |  |  |  |
| 38 | .015 | .039 | 99.960 |  |  |  |  |  |  |
| 39 | .010 | .024 | 99.984 |  |  |  |  |  |  |
| 40 | .006 | .016 | 100.000 |  |  |  |  |  |  |
